# The international effort: building the bridge for Translational Medicine: Report of the 1st International Conference of Translational Medicine (ICTM)

**DOI:** 10.1186/2001-1326-1-15

**Published:** 2012-08-14

**Authors:** Xiaoming Chen, Roland Andersson, William CS Cho, David Christiani, Richard Coico, Jeffery Drazen, Markus Ege, Thomas Fehniger, Hongwei Gao, Kunlin Jin, Michael N Liebman, Elena Lopez, Giuseppe Marraro, Gyorgy Marko-Varga, Francesco M Marincola, Laurentiu M Popescu, Claudio Spada, Aamir Shahzad, Ena Wang, Wei Wang, Xiangdong Wang, Yong-Xiao Wang, Jinglin Xia, Jia Qu

**Affiliations:** 1 The first Affiliated Hospital of Wenzhou Medical College, Wenzhou, China; 2 Department of Surgery, Clinical Sciences, Lund University Hospital, Lund, Sweden; 3 Department of Clinical Oncology, Queen Elizabeth Hospital, Kowloon, SAR, Hong Kong; 4 Department of Environmental Genetics, Harvard School of Public Health, Massachusetts General Hospital, Boston, MA, USA; 5 Department of Cell Biology and Medicine, State University of New York-Downstate Medical Center, Brooklyn, NY, USA; 6 Department of Environmental Health, Harvard Medical School, Boston, USA; 7 University of Munich, Germany Children’s Hospital, Munich, Germany; 8 Tallinn University Of Technology (Estonia), Lund, Sweden; 9 Department of Anesthesiology, Perioperative & Pain Medicine, Harvard Medical School, Boston, USA; 10 Department of Pharmacology and Neuroscience, University of North Texas, Fort Worth, USA; 11 Strategic Medicine, Inc (PA, USA) and Strategic Medicine, the Hague, BV, Netherlands; 12 Centro de Investigación i+12 del Hospital Universitario 12 de Octubre, Avda de Córdoba s/n, Madrid, Spain; 13 Department, Pediatric Intensive Care Unit, Fatebenefratelli & Ophthalmiatric Hospital Milan, Milan, Italy; 14 Deptartment of Measurement Technology and Industrial Electrical Engineering, Lund University, Lund, Sweden; 15 Infectious Disease and Immunogenetics Section (IDIS), Department of Transfusion Medicine, Clinical Center and Center for Human Immunology (CHI), NIH, Bethesda MD, USA; 16 Department of Advanced Studies, National Institute of Pathology, Bucharest, Romania; 17 MISM Science & Technology Consulting - SCConsulting, Milan, Italy; 18 Max F. Perutz Laboratories (MFPL) University of Vienna, Vienna, Austria; 19 Edith Cowan University, Perth, Australia, Chinese Academy of Sciences and Capital Medical University, Beijing, China; 20 Biomedical Research Center, Fudan University Zhongshan Hospital, Zhongshan, China; 21 Center of Cardiovascular Sciences, Albany Medical College, Albany, NY, USA; 22 Hepatic Oncology, Zhongshan Hospital, Fudan University, Shanghai, China; 23 School of Ophthalmology and Optometry, Wenzhou Medical College, Wenzhou, China

**Keywords:** Translational Medicine (TM), International Society for Translational Medicine (ISTM), the International Conference on Translational Medicine (ICTM), Biomarkers, Biobank globalization and networking

## Abstract

**Background:**

Supported by the International Society for Translational Medicine (ISTM), Wenzhou Medical College and the First Affiliated Hospital of Wenzhou Medical College, the International Conference on Translational Medicine (ICTM) was held on October 22–23, 2011 in Wenzhou, China. Nearly 800 registrants attended the meeting, primarily representing institutes and hospitals in Europe, The United States of America, And Asia, and China. The meeting was chaired and organized by Dr. Xiangdong Wang, Xiaoming Chen, Richard Coico, Jeffrey M. Drazen, Richard Horton, Francesco M. Marincola, Laurentiu M. Popescu, Jia Qu and Aamir Shahzad.

**Findings:**

The meeting focused on the communication of the need to foster translational medicine (TM) by building and broadening bridges between basic research and clinical studies at the international level. The meeting included distinguished TM experts from academia, the pharmaceutical and diagnostics industries, government agencies, regulators, and clinicians and provided the opportunity to identify shared interests and efforts for collaborative approaches utilizing cutting edge technologies, innovative approaches and novel therapeutic interventions. The meeting defined the concept of TM in its two-way operational scheme and emphasized the need for bed to bench efforts based directly on clinical observation.

**Conclusions:**

It was the meeting participants’ realization that the shared main goals of TM include breaking the separation between clinic practice and basic research, establishing positive feedback by understanding the basis of expected and unexpected clinical outcomes and accelerating basic research relevant to human suffering. The primary objectives of the meeting were two-fold: to accelerate the two-way translation by informing the participants representing the different disciplines about the state of art activities around TM approaches; and to identify areas that need to be supported by redirecting limited resources as well as identifying new sources of funding. This report summarizes key concepts presented during the meeting representing the state-of-art translational research and salient aspects of the ensuing discussions.

## Findings

### Introduction

It is becoming increasingly clear from the current state of the science that Translational Medicine (TM) should increase its international efforts. Advances in technology and medicine produced an unprecedented wealth of data which extends our understanding of human biology to a completely new dimension. Placing these data into useful context for developing better clinical practice is complicated by the sheer size of datasets being generated and the limited set of tools for translating this information into useful products such as biomarkers, targets for therapeutic intervention, and safe and effective drugs or diagnostic compounds. In the last decade much resource has been dedicated to providing useful biomarkers that can aid in clinical decision making and patient management.

However, among the numerous biomarkers identified, only a few have undergone extensive validation. In the same fashion, drug discovery is facing an even less rewarding reality as few originally promising products eventually pass the standards demanded for approval after long clinical trials. Meanwhile, disease mortality and morbidity have barely changed over the years, other than that which results from early screening programs that support early detection and intervention. Thus, the efforts and resources consumed on the generation of experimental data has been disproportional to the obtained results aimed at the improvement of patient suffering. It is clear that while current technologies have almost limitless potential, barriers such as inadequate study design, limited standardization and cross-validation among laboratories, suboptimal comparability of data, missing globalized efforts and inefficient utilization of limited resources remain major road blocks. The institution of an interactive consortium for a globalized biorepository, high throughput and multidimensional technologies, sophisticated experimental and clinical study design, strong bioinformatics approaches, and harmonization of studies through the implementation of standard operative procedures with voluntary participation might provide cost-effective solutions.

### The Evolution of Translational Medicine (TM)

Dr. Richard Coico, Ph.D., Professor of Cell Biology and Medicine, State University of New York-Downstate Medical Center, Brooklyn, NY pointed out that although TM is considered an emerging new field, in reality, elements of it have been practiced for millennia. Its evolution can be traced back to ancient Egypt (c. 2600 BC) when philosophers serving as clinicians, observed and promoted the use of various dietary and topical remedies for disease (e.g. fruits, linseed oil). They were also the first to demonstrate the antiseptic properties of Willow leaves and bark. These and many other examples of the successful translation of simple observations into useful clinical applications demonstrate how rudimentary healing practices became the standard of care. In the middle ages, Avicenna (c. 1030) wrote *The Canon of Medicine* which established, for the first time, the legitimacy of experimental medicine and the importance of evidence-based medicine. The *Canon* was used as a standard textbook in Muslim and European universities until the 18^th^ century. During the same period, discovery of the therapeutic utility of digitalis in treating heart disease was made by the English botanist and physician, William Withering, ushering in what is considered the beginning of modern therapeutics. These and countless other translational medicine benchmarks in the 18^th^-early 20^th^ centuries were obtained long before the tools of molecular biology, computational biology, genomics, proteomics, etc. and the rules of ethical treatment of human subjects for research, animal welfare, and bio-safety were available. The complexity of translating observations to clinical applications has progressively increased throughout the centuries due to ethical and practical concerns. However, the goals remain the same. The ancestral achievements in medical understanding will need to be matched in the 21st century by exploiting new and novel technologies, and by improving study design and coordinating cross-discipline activities which, in ancient times, would have been considered magical.

#### Translational medicine-the paradigm shift

Dr. Francesco M. Marincola, Editor-in-Chief of the *Journal of Translational Medicine*, President Elect of ISTM and vice-President of SITC, Chief of the Infectious Disease and Immuongenetics Section (IDIS), Department of Transfusion Medicine (DTM), Clinical Center (CC), National Institutes of Health (NIH) has been a strong advocate for translational medicine for the last decade. He echoed that the TM is not a novel concept and has many different definitions with the same intention. However, TM emphasizes the modern need to increase the efficiency of the process over various hurdles that hamper the translation from oberservation to clinical testing. Fewer than 5% of new agents tested in clinical trials reach regulatory approval and less than 1% will turn into successful products [1-3]. Therefore, the novelty of TM lays on redirecting funding resources to accelerate the transition by catalyzing research relevant to humans for the ultimate benefit of patients. It has been almost a decade since he suggested that translational medicine is a two-way road where the predominant emphasis on the bench-to-bedside direction should be balanced by the bedside-to-bench one. He proposed that attention to clinical relevance should play a leading role in framing scientific questions according to human actuality. He highlighted that hypotheses should first emerge from careful observation of events relevant to human diseases. Such observations should be based on skillful studies performed in human subjects, at relevant time points and at the site where the patho-physiological process and its perturbation induced by treatment is evolving. Translational research is caught in a vicious cycle whereby: 1. the enterprise is based on a less than solid understanding of human pathophysiology particularly when complex, multi-factorial diseases are confronted; 2. scientists “hypothesize” based on individual experiences as to relevance to human disease based on the comfort zone of their knowledge rather than an in depth understanding of human disease; 3. pre-clinical models are generated, which are, in turn, poor predictors of effectiveness in humans because they do not take into account the salient aspect of human pathophysiology and its fundamentally unpredictable nature. Hence, only by identifying, from thousands of hypotheses, the patho-physiological common themes that actually occur in humans, scientists will be likely to develop therapeutic strategies likely to work in most if not all patients.

Dr. Michael N. Liebman, Managing Director, Strategic Medicine, Inc. (PA, USA), discussed the issues of stratification and personalization in medicine within the context of translational medicine. He clarified conceptual differences among translational, personalized and stratified medicine. In agreement with Dr. Marincola, TM should not only start in the lab and expect to translate results into the clinic but the bedside-to-bench-to-bedside approach should be implemented. TM has focused more on the transition from basic research to clinical use while personalized medicine and stratified medicine have focused on stratifying the patient utilizing genetic markers to tailor individual treatments for specific groups of patients. His emphasis was on examining the stratification of the disease, not just the patient,, and that although genetics may determine an individual’s potential benefit from treatment, it is critical to include lifetime influences of environmental exposure and lifestyle experiences which modulate the impact of genetics, both positively and negatively. He advocated that human disease is not a state as we frequently refer to “disease state” but rather a process which is complicated by numerous factors such as genetic predisposition, acquired predispositions to disease, co-morbidity, length of disease since diagnosis, treatment history, diverse response to a given therapy and other yet unspecified modifiers. Integration and research into the role of those factors will likely yield greater success in improving patient care and developing new diagnostics and treatments than the ongoing development of the new technologies which are constantly being introduced to improve speed and efficiency in both basic research and the developmental phase of drug development. Therefore, disease stratification must be based on information derived from an expanded personal health record for the patient, along with the use of advanced technology, and comprehensive clinic testing followed by the development and application of sophisticated data mining and bioinformatics analysis [4]. He presented examples of breast cancer patient stratification by a modeling approach whereby cancer patients were stratified according to histology, tumor progression pattern, TNM staging normalized according to peri-menopausal to post menopausal status. This approach included pathway and network analysis based on regulation of gene transcription and genetic polymorphism. He also summarized hurdles confronting clinical investigation that include high cost, non standardized patient selection criteria, and inadequate comprehensive clinical information before enrollment and inadequate hypothesis development.

### International effort in TM

Dr. Jia Qu, School of Ophthalmology and Optometry, Wenzhou Medical College, China, summarized worldwide efforts in TM evidenced by the launch of Clinical and Translation Research Centers in more than 30 medical colleges, the establishment of Clinical and Translational Science Awards and the National Center for Advancing Translational Sciences (NCATS) with $575 million Government funding at NIH in the United States. In Europe, 6 billion Euros has invested for translational research. UK will spend 450 million pounds to build translation medicine centers within the next 5 years. However, TM in China is challenged by limited financial support, relative weak support from basic research and a publication-focused system rather than aimed at patient benefit. Based on his success in translation from the research development of optical coherence tomography (OCT) to ophthalmic medicine application, he proposed that team work and broad collaboration is essential for advance and success in TM.

As the awareness and excitement of applying translational medicine increase worldwide, journals and articles focused on TM or related to TM are proportionally swelling accordingly. Dr. Jeffrey M. Drazen, Editor-in-Chief of the *New England Journal of Medicine* exemplified journal publication process and its sensitivity to translational science emphasizing that the decision making in the selection of manuscripts for publication is based predominantly on their influence on clinic practice. In 2010, more than 25% of original research articles published by the *NEJM* had a translational scope. There were a significant number of submissions from China in 2010, but only minorities of the submitted manuscripts were accepted for publication due to either substandard scientific quality or poor writing due to language issues. Nevertheless, he encouraged the meeting participants to aim for high impact publications by giving several examples of recently published manuscripts adopting well-designed study strategies addressing high impact clinical question. *Dr Drazen welcomed inquiries from perspective submitters to ask for advice from the Editorial Office prior to submission. He also highlighted several new methods in journalism that are being launched to include electronic media, such as videos of research studies presented as stories, and for the need to make scientific advancements more easily understood within society.*

#### Complexity of translational research

Fundamental strides in the understanding of the molecular basis of disease have been made in the last decade thanks to observational studies performed at relevant time points in humans. The advance of technology and their application revealed the complexity of human biology in pathophysiological conditions. Dr. Ena Wang, Director of Molecular Sciences, Department of Transfusion Medicine, Clinical Center, and Associated Director, trans-NIH Center for Human Immunology, National Institutes of Health illustrated the impact of genetic polymorphisms, global transcriptional regulation, epigenetic regulation, environmental influence and preexisting conditions in the interpretation of human biology. She proposed that a system biology approach with multidimensional analysis including all possible variants should be considered when dissecting human pathology. The low yield of relevant information derived from clinical studies results mainly from the insufficient clinical and patient information collection, minimal stratification of variables contributing to a given condition, biased selection of patient population and non standardized research procedures. She presented examples of using system biology to study the significance of transcriptional signatures observed in pre-treatment biopsies as predictive of responsiveness to immune therapy. By comparing transcriptional signatures observable during and after immune therapy a convergent characterization from chronic to acute inflammation switch was observed. The switch not only can lead to tumor rejection but more in general to all forms of immune-mediated tissue destruction processes including flares of autoimmunity, clearance of pathogen-infected cells, allograft rejection and graft versus host disease. This observation suggests that each model system has its own idiosyncrasies but, at the same time, commonalities dictate the final outcome of rejection. Understanding of the basic mechanisms of tissue rejection may guide the development of novel therapeutic strategies. Even more importantly, identifying the mechanisms that lead to this final common pathway in individual tumors may define a better rational for targeted therapies. These are good examples of evidence-based clinically relevant research that has generated hypotheses that could now be pursued at the bench side.

### Biomarker discovery

Biomarkers comprise a very broad concept. The term biomarker usually refers to any biological measurement that provides information regarding disease progression, relationship with treatment, safety and can be used for decision making in clinical or experimental applications. Depending upon the source, time, condition and possible application, biomarkers can be categorized into 1) translational biomarkers: a biomarker that can be applied in both a preclinical and clinical setting; 2) disease biomarkers: biomarkers that relate to a clinical outcome or measure of disease; 3) efficacy biomarkers: a biomarker that reflects beneficial effect of a given treatment; 4) staging biomarkers: a biomarker that distinguishes between different stages of a chronic disorder; 5) surrogate biomarkers: a biomarker that is regarded as a valid substitute for a clinical outcomes measure; 6) toxicity/safety biomarkers: a biomarker that reports a toxicological effect of a drug on an in vitro or in vivo system; 7) mechanistic biomarkers: a biomarker that reports activity downstream of signaling pathway; and 8) target biomarkers: a biomarker that reports interaction of the drug with its target. Dr. Xiangdong Wang, Professor of Medicine and Molecular Bioscience, Director of the Biomedical Research Center, Fudan University Zhongshan Hospital, China, presented strategies in biomarker discovery with different objectives in clinic application and in biomedical research. Irrespective the great strides being made in biomarker identification, there are as yet unmet needs in developing standards and guidelines that define toxicology, clinical phenotyping, , dosage decision making, treatment attrition decisions, and patient stratification. There are also pressing needs to improve in early disease diagnosis, health care system management and integration, new drug effect validation, medical instrumentation, and diagnostic tests that comply with regulatory standards. Dr Wang also emphasized that clinical bioinformatics that provided systematic information associated with patients has been under appreciated and is one key step towards translational research.

### Definition of “healthy status”

One critical premise of disease-related biomarkers is the definition of their counterpart, normality. Contrary to pre-clinical models that can be carefully tailored to meet scientific need and preferred outcomes, the essence of human disease involves heterogeneity in clinical presentation and with a complex biological basis that is not easily controlled for all possible models of behavior in a population of subjects. A full characterization of the consistent parameters that can define the predicted behavior of disease processes developing in previously healthy subjects is as yet not attainable. Self proclaimed normal status may not represent health because asymptomatic subjects may carry chronic diseases or diseases at their early stage such as seen in many cancer types. Professor Wei Wang, MD, PhD, from Edith Cowan University, Perth, Australia, Chinese Academy of Sciences and Capital Medical University, Beijing, China, and Professor Toshio Fujioka MD, PhD from Oita University, Oita, Japan exemplified the characterization of the suboptimal health status (SHS) which represents a new public health problem in a population with ambiguous health complaints such as general weakness. They applied clinical informatics approaches and developed a questionnaire for measuring SHS. The validity and reliability of this approach were evaluated in a small pilot study and then in a cross-sectional study of 3,000 individuals. The final questionnaire segregated into a score (SHSQ-25) which could significantly several abnormal conditions and could be used in the general population [5-7].

### Epigenetic biomarkers

Epigenetic interactions occur at many levels and result in changes in the genome by a variety of mechanisms that are driven by including transcriptomic, metabolomic or proteomic interactions that play a major role in determining phenotypes beyond genetic background. Among epigenetic components, micro RNA (miR), functions as regulatory RNA target transcripts that lead to protein translation inhibition or messenger RNA degradation. Variable expression of miRs has been associated with diverse vascular diseases [8-11]. Recent studies have demonstrated that miRNAs are aberrantly expressed in proliferative vascular disease, cardiac hypertrophy, heart failure, and ischemic heart disease. Chunxiang (Kevin) Zhang MD, PhD, Director of RNA& Cardiovascular Research Laboratory, and Vice Chairman for Research at Department of Anesthesiology, New Jersey Medical School, UMDNJ described his work on miR function in association with Cardiovascular Diseases. By miR array profiling, his group observed that miR-145 is highly expressed in normal arteries but aberrantly expressed in stenotic arteries in experimental animal model and as well as in human atherosclerotic aortas with neointimal growth. Based on the observation that abundant release of miR-1 from necrotic cardiac myocytes following acute myocardial infarction in animal model, they identified that miR-1 levels increase 100 times following AMI in humans. Therefore, miR-1 could be a potentially novel diagnostic marker for AMI patient.

Dr. William CS Cho, , PhD, CSci, FIBMS, Chief Editor of *Frontiers in Non-Coding RNA*, *Department of Clinical Oncology, Queen Elizabeth Hospital, Hong Kong SAR* studied miRs as biomarkers in lung cancer. He discovered that miR-145 plays an important role in inhibiting lung cancer proliferation by targeting EGFR and NUDT1. Restoring miR-145 expression in vitro in EGFR mutated lung cancer cell line could significantly reduce cell proliferation. In patients with NSCLC, EGFR mutations are strongly associated with EGFR-tyrosine kinase inhibitors (TKI) sensitivity. This finding suggests that it may be possible to individualize EGFR-TKI treatment for lung adenocarcinoma patients based on EGFR mutation [12]. However, miR may represent promising markers, their application in the clinics is challenged by tissue biopsies accessibility. As an alternative, the identification of circulating miRNAs may represent a valuable strategy for noninvasive early diagnosis and follow-up investigations [13].

### Protein biomarkers

Protein biomarkers have been used clinically as a standardized laboratory reference to diagnose and differentiate the status of both health and disease. These measurements play a significant role in clinical decision making, both as comparators with normal ranges and activities and as monitors of dysfunctional physiologic and metabolic processes in cells located in specific organ systems [14]. Prof. Thomas Fehniger, Institute of Clinical Medicine, Tallinn University of Technology, Tallinn, Estonia, exemplified the importance of protein structure and function in relation to human health and disease. He emphasized the importance of linking panels of disease related biomarkers with histopathological changes observed in diseased tissues.

Clinical proteomics is an important component of TM. However, protein biomarker discovery is challenged by detection sensitivity and low abundance of many functional proteins involved in disease processes. Intracellular protein content exhibits 9-orders of magnitude in range of concentration. Depending on specific protein abundance, the required number of cells to detect a given substrate can range from 6e3 cell with 1e6 copy of protein to 6e8 cell with 10 copy of protein. As a result, proteins discovered by the HUPO’s plasma proteome project are largely distributed in the concentration above ug/ml range while clinically used biomarkers are mainly in ng to pg range [15]. Therefore, identifying proteins in the right context remains an important obstacle to overcome. He brought attention to the fact that in the current status, there is a gap between proteomic biomarker discovery and their quantification. He urged the need for well characterized patients and samples for biomarker discovery; the need to establish reference standardizations in measurements and criteria for validation in population based studies in global settings.

To overcome the low abundance of functional protein, Aamir Shahzad, MD, Editor in chief, Translational Biomedicine Journal (TBM), University of Vienna, Austria applied fluorescence correlation spectroscopy (FCS) to study interactions among bio-molecules expressed at extremely low-concentration in solution. FCS was able to measure diffusion time and the average number of fluorescent molecules passing through the open detection in 1ul volume. The technology can detect proteins directly without incubation nor enzymatic reaction and allows the study of complex formation between a small fluorescently labeled and a large unlabeled molecule. In addition of the sensitivity and reproducibility, the test results can be obtained in 15 min which make it a potential tool for clinical application.

Post-translational modifications (PTM) are a major challenge in protein biomarker identification. Reversible protein phosphorylation, an important type of cellular regulation, controls many biological processes such as cell growth, differentiation, invasion, metastasis and apoptosis. Abnormal protein phosphorylation can be the cause or consequence of many diseases such as cancer. The deregulation of protein kinase activity with its consequent change in protein phosphorylation states has been associated with the onset of tumor formation and cancer progression. The importance of protein kinase-regulated signal transduction pathways in human cancer has led to the development of drugs that inhibit protein kinases at the apex or intermediary levels of these pathways. Phosphoproteomic analysis of these signaling pathways could provide important insights into the operation and connectivity of these pathways and can facilitate the identification of the best targets for cancer therapies. Elena López, PhD from Hospital Universitario12 de Octubre, Spain, updated the current phosphoproteomic approaches by comparing advanced strategies. Though no standard procedure has been established, Dr. López proposed that the combination of multi-stage collision-induced dissociation (CID) fragmentation, the development of electron transfer dissociation (ETD) and electron capture ECD fragmentation of peptides in mass spectrometry (MS) with phosphor-enrichments may be the method of choice to study protein post-translational modifications. MS-based phosphor-proteomics tools are critical to understand the structure and dynamics of signaling that engages and migrates through the entire proteome. These PTMs ultimately give rise to the emergent functions of cells in sequence, space and time. She also summarized various approaches used for the analysis of the phosphor-proteome in general and protein kinases in particular, highlighting key cancer phosphor-proteomic studies. Different proteomic and bioinformatics strategies need to be combined to achieve good phosphor-peptide quantitative-protein studies. From the point of view of the so-called “personalized medicine”, bioinformatics studies of reversible phosphorylation in proteins will allow the generation of models for protein-protein interaction at the atomic level taking into account each particular protein sequence. Molecular dynamic analysis of those interactions will allow the modification of the 3D computer models obtaining virtual structures tailored to individual patients [16-18].

### Pre-clinical Studies

Several pre-clinical models were presented as examples of bench-to-bed efforts. It is well known that pulmonary arterial hypertension (PAH) is a prevalent disease with a high mortality rate, and often afflicts patients with chronic obstructive pulmonary disease (COPD) and other chronic lung diseases as well as high altitude residents due to chronic hypoxic cellular responses. However, the molecular mechanisms underlying PAH remain unclear, and current therapeutic options remain limited. Yong-Xiao Wang from the Center of Cardiovascular Sciences, Albany Medical College, Albany, New York, USA described the role of ryanodine receptors/calcium release channels (RyRs) in pulmonary artery smooth muscle cells (PASMCs) in the development of hypoxic PAH. He reported that RyR activity, associated calcium release and contraction were largely increased in PASMCs from mice with hypoxic PAH. The increased activity of RyRs was confirmed in PASMCs from COPD patients with PAH. Pharmacological and genetic blockade of RyRs almost completely prevented hypoxic PAH in mice. These exciting results provide the first evidence that RyR calcium signaling plays an essential role in the development of hypoxic PAH, and also suggest RyR antagonists that are available and even used in clinic may become potentially new and effective therapeutics to treat PAH and other relevant pulmonary vascular diseases in the future.

To explore the mechanism of acute lung injury, Hongwei Gao, Center for Experimental Therapeutics and Reperfusion Injury, Brigham and Women's Hospital, Department of Anesthesiology, Perioperative & Pain Medicine, Harvard Medical School, Boston, described how Stat3 is activated in alveolar macrophages and whole lung extracts in the IgG immune complex-induced lung injury model. This could be dramatically suppressed by blockade of C5a in the lung or depletion of either neutrophils or alveolar macrophages. Treatment of mice with an adenoviral vector expressing a dominant-negative Stat3 isoform (Ad-Stat3-EVA) eliminated IgG immune complex-induced DNA binding activity of Stat3 in the lung and led to a significant decrease in the contents of neutrophils, inflammatory cytokines (TNF-α and IL-6), chemokines (KC, MIP-1α, MIP-1β), and complement component C5a in bronchoalveolar lavage fluids compared with control. This suggested that Ad-Stat3-EVA might affect neutrophil adhesion to the lung vascular endothelium and/or their subsequent transmigration by modulation of adhesion pathway(s).

### Gene-environment interactions in pathogenesis

In the past decade, great effort has been made to explore the genetic background of humans and its association with disease. This effort led to the completion of Human Genome Project and the Hapmap project. Ongoing international efforts include the Cancer Genome Atlas, genetic association information network, genome wide association, gene and environment initiative, and the 1000 genome study. Linkage analysis of single nucleotide polymorphism and whole genome-wide association has been applied to the study of specific diseases which has increased our understanding of the contribution of genetics to pathogenesis. Genetic polymorphisms are the basis of diverse human phenotypes, disease susceptibility, prognosis and responsiveness to treatment. Though genetic traits play a pivotal role, they could be influenced by environment factors. Markus Ege, MD MPH, University of Munich, Germany Children’s Hospital, Munich, Germany gave an example of gene and environment interactions in asthma and allergy. Polymorphisms of a number of genes have been consistently related to asthma [19,20]. These genes are involved in pathways of the innate and adaptive immunity and in epithelial barrier functions [20]. However, among children who carry the identified polymorphisms, only a minority developed asthma. Paradoxically, asthmatics can carry a low genetic asthma risk while healthy individuals can carry high genetic asthma risk. Those cases of asthma occurring without a genetic predisposition could be due to environmental exposures related to asthma such as tobacco smoking [21]. A trend was documented of increased prevalence of asthma is in areas of high industrialization and pollution. As the isolated assessment of genetic and environmental determinants cannot explain causation of asthma alone, he hypothesized that the enhancement of genetic predisposition by environmental determinants might provide a better explanation. His group applied comprehensive assessment of gene/environment interactions using a genome wide approach with a statistical method that controls for multiple testing [22]. Despite adequate statistical power only few weak novel interactions were detected suggesting that gene/environment interactions might be even more complex and should be sought at different levels comprising global gene expression and analysis of epigenetic modifications. Alternatively the genetic and environmental effects in this population were so strong that they did not modify each other.

Notwithstanding the importance of environmental factors in asthma, David C. Christiani, MD, MPH,MS Elkan Blout Professor of Environmental Genetics Harvard School of Public Health, Massachusetts General Hospital, Boston, Massachusetts, furthered his study in how genetics and environment interact to increase lung cancer risk. Susceptibility of lung cancer at different time points along the natural history of an individual depends on many factors such as exposure to smoking, dose of smoking, lung structure and function alteration and disease progression. He reported how his group investigated how DNA repair function gene variants interact with smoking to increase lung cancer risk. Among those genes, ERCC2 Asp312Asn, XRCC1 Arg399Gln, ERCC1 C8092 genotype shows a significant impact from years of smoking, going from protective to non-protective as indicated by inversed correlation of adjusted odds ratio and pack-years of smoking. A genome wide association scan (GWAS) conducted by multi centers identified a susceptible locus (15q25) for lung cancer [23] which accounts only for 4% of lung cancer risk. To interrogate whether variants on the 15q25.1 region affect lung cancer through nicotine dependence and are associated with smoking or through other pathways, they applied novel methodology for analysis of 1836 lung cancer patients and 1452 controls and found evidence of an effect mediated through smoking. In addition, a positive correlation between cumulative cigarette smoking and CNV were observed which provides insight into the genetic toxicology of tobacco smoke. Therefore, Dr. Christiani concluded that common diseases such as lung cancer likely result from genetic and environmental disturbances in entire networks of genes rather than in a single, or even a several genes.

#### Application of translational medicine

Stroke is one of the leading causes of mortality and disability in the world. Stroke survivors are often left with permanent neurological deficit resulting from the loss of neurons, which severely impacts their quality of life. From 1955–2000, 49 neuro-protective agents for stroke therapy have been tested in 21,445 patients. However, none of the tested agents were approved for clinical application [24]. Current treatment options are limited. Dr. Kunlin Jin, Tenured Professor at the Department of Pharmacology and Neuroscience, University of North Texas Health Science Center at Fort Worth, Texas, USA, presented recent advances in stem cell-based therapy for chronic stroke. His group and others have shown in animal models that neural precursor cells transplanted into effected regions of the brain can differentiate into neurons, and have the potential to improve functional outcome after stroke. To regenerate damaged brain tissue in the infarction cavity after stroke, they applied tissue engineering approaches using neural stem cells in scaffolds grafted in the cavity. This is based on the hypothesis that the scaffolds may not only provide the necessary biomechanical support for cells, guide the gross shape and size of the regenerated tissue into forming three-dimensional tissues, but also provide artificial stem cell niches necessary for stem cell growth. Additionally, scaffolds can serve as biochemical signals that influence cell adhesion, migration, proliferation, differentiation and functions. His group successfully experimented with *in situ* tissue engineering using a combination of injectable and biodegradable scaffolds and hESC-derived neural stem cells to mouse brain cavity caused by experimental stroke. This result suggests that this approach has great potential as a powerful new therapy for stroke in human.

Prof Xiaokun Li Phd, School of Pharmacy, Wenzhou Medical College described how fibroblast growth factors (FGF) plays a critical role in cell division, development and wound healing via activation of growth factor receptor and initiation of a downstream cascade of signal transduction, and gene activation. Experimental data have shown that a single amino acid substitution at position 78 and 96 of the protein [25]or N-terminal removal [26] makes the mutated FGF more stable without impacting its biological function. PEGylation modification of the recombinant FGF21 makes it not only stable but long lasting as an anti-diabetic drug [27,28] and a promising drug for wound healing according to animal models. With a chitosan-cross-linked collagen sponge (CCCS) as scaffold that contains recombinant human aFGF (CCCS/FGF), accelerated diabetic wound healing and corneal epithelial wound healing were observed. An FGF analog (aFGF) is being tested in a phase II clinical trial that is suggesting significant improvement of diabetes wound healing and burn healing. Products were derived from this study including bFGF eye drop (Cornea recover), membrane, gel, freeze-dry powder, collagen sponge and spray for clinical use.

Professor Lawrence M. Popescu, Department of Advanced Studies, National Institute of Pathology, Bucharest, Romania, presented the discovery and characterization of interstitial cells – Telocytes (TCs) which have been found in cavitary and non-cavitary organs of humans and mammalians. TCs have a small cell body with specific prolongations named telopodes (Tps). TCs can modify neighbor cells transcriptional activity by shedding vesicles and/or exosomes facilitating precursor cells towards maturation and their integration into the microscopic architecture of tissues. This function is supported by the experimental observation that TC are directly and indirectly involved in neo-angiogenesis after myocardial infarction suggesting a possible application in regenerative medicine.

#### The need for guidelines and standardization

The past decade, basic research has decoded numerous unknown entities relevant to biology and accelerated our understanding of physiology and pathology. There are far more basic researchers and publications based on *in vitro* or experimental models which are more easily controllable and as a consequence more easily funded and published. However, among candidate drugs successfully tested in animals, very few withstand the extension to humans due to fundamental differences in genetic makeup of humans, their heterogeneity and the heterogeneity of their diseases and the impact of the easily controllable genetics within inbred animals. There is also a high level of complexity and diversity in human genetic and genomic compositions that produce biology that cannot be modeled experimentally or foreseen in the results generated in animal studies. It is important to emphasize that TM should foster bed-to-bench effort to provide better understanding of human pathology and understanding the reasons for failure of current standard or experimental therapies. The first step toward study humans is to collect samples which preserve the maximum in vivo information and can be used by diverse yet available cutting edge technologies.

Dr. György Marko-Varga, European Editor of the *Journal of Proteome Research*, ACS, and President of The European Proteomics Society (http://www.EuPA.org), Division of Clinical Protein Science & Imaging, Biomedical Center, Dept. of Measurement Technology and Industrial Electrical Engineering, Lund University, Sweden, urged the great importance of the establishment of a global biobank to speed up the discovery and development of new drugs and protein biomarker diagnostics. Biobanks are providing patient benefit by allowing large scale screening and generating large database repositories. Biobanks are a major resource for scientists to access unique patient samples for medical research. Many studies within clinical proteomics utilize stored samples contained in Biobanks to measure specific end points. It is envisioned by all participating stakeholders and ICTM participants that the biobank initiatives will become the future gateway to discover new frontiers within life science, patient care and catalyzing the two way road of TM [29]. Biobank globalization and networking by combining and sharing precious samples and information associated with the sample can empower large joint studies to tackle major biological question with limited resources and eliminate less meaningful small repetitive study due to the complexity of human biology and heterogeneity of patients. Standardization of quality control of samples being processed for storage as well as retrieval of stored samples is important goals in order to support the development of diagnostic tools such as biomarkers. Today we have not yet achieved consensus on how to collect, manage, and build biobank archives in order to reach goals where these efforts are translated into value for the patient.

Effort in developing guidelines for more effective and safe therapy should also be enhanced. Dr. Claudio Spada, MISM Science & Technology Consulting - SCConsulting, Milan, Italy pointed out that Guidelines developed in 2002 required significant changes based on new information and advances in medicine. He updated, in 2007, the guidelines for treatment of sepsis compared with 2002 guidelines. As new developments in diagnosis and promising therapies tested in animals and humans will emerge, they will need to be validated in large clinical trials to confirm the safety and efficacy of their use in septic patients.

Roland Andersson, MD, PhD from Department of Surgery, Clinical Sciences, Lund University Hospital, Sweden updated the epidemiology and available therapy for pancreatic cancer. He discussed the challenges in lack of biomarkers for early diagnosis and prognosis [30], although this field has been actively explored. Numerous therapeutic agents and antibodies have been developed targeting IGF-1R, EGRR and VEGFR signaling pathways with intention to block cancer cell proliferation, angiogenesis or revert drug resistance [31,32]. The improved survival of pancreatic patients has not significantly encouraged broader investigation of tumor biology facilitated by microenvironment. In the improvement of early diagnosis of pancreatic cancer, proteomic application using serum/plasma has revealed over expression of Alpha-1B-glycoprotein precursor, Anterior gradient 2, Apolipoprotein C-I, DJ-1, Fibrinogen β chain, HSP27 and under expression of Apolipoprotein A-II, Caldecrin, CXCL7. Dr. Andersson pointed out that although a multitude of investigational biomarkers have been identified; translating into routine clinical use has been slow. It was realized that standardization in analysis techniques and better reporting will improve the translation of biomarkers. New guidelines for diagnostic biomarkers [33] and for prognostic markers (REporting recommendations for tumor MARKer prognostic studies [34] are available and should be followed closely.

### Mass spectrometry imaging

Dr. György Marko-Varga, gave a presentation on a new technology area where it is possible to localize a drug compound with a resolving power within a single cell. Readouts that define the physiological distributions of drugs in tissues are an unmet challenge and at best imprecise. It is also needed in order to understand both the pharmacokinetic and pharmacodynamic properties associated with efficacy. Drug therapy is often directed to specific organ and tissue compartments where the mode of action of the compound affects specifically targeted biological processes. However, the direct measurement of drug uptake in terms of a time kinetic and concentrations attained at the local sites has not been readily available as a clinical index for most drugs. It is feasible to follow the unlabeled drugs within specific organ and tissue compartments on a platform that applies MALDI imaging mass spectrometry to tissue sections characterized with high definition histology. Moreover, the simultaneous global definition of molecular ion signatures localized within 2-D tissue space provides accurate assignment of ion identities within histological landmarks, providing context to dynamic biological processes occurring at sites of drug presence. He reported on a recently performed proof-of-principle study that tested the power of applying MALDI-MSI to demonstrate the qualitative drug distribution of inhaled bronchodilators in COPD patients. The study mapped the occurrence of the muscarinic receptor antagonist ipratropium, within human bronchial biopsies obtained by fiber optic bronchoscopy shortly after dosing exposure. This is the first reported study in man, at a resolving power of 30 μm, of drug localization within organ microenvironments using normal clinical dosing schemes and standardized laboratory measurement [35]. He also emphasized that MALDI-MSI approaches will play an increasingly important role in both drug development in the optimization of compounds and in clinical efficacy testing.

## Competing interests

The authors declare that they have no competing interests.

## Authors’ contributions

XC, JQ, XW RA and FMM are Key meeting organizers. WCSC, DC, J D, ME, HG, KJ, MNL, GM, GMV, FMM, LMP, CS, AS, EW, WW, XW, YXW, JX and JQ contributed in meeting presentation and meeting report revision. EW, GMV and FMM contributed in meeting report drafting. All authors read and approved the final manuscript.

## References

[B1] Contopoulos-Ioannidis DG Ntzani E Ioannidis JP Translation of highly promising basic science research into clinical applications Am J Med 2003 114 6 477 484 10.1016/S0002-9343(03)00013-5 12731504

[B2] Ioannidis JP Materializing research promises: opportunities, priorities and conflicts in translational medicine J Transl Med 2004 2 1 5 10.1186/1479-5876-2-5 14754464 PMC343300

[B3] Contopoulos-Ioannidis DG Alexiou GA Gouvias TC Ioannidis JP Medicine. Life cycle of translational research for medical interventions Science 2008 321 5894 1298 1299 10.1126/science.1160622 18772421

[B4] Liebman MN Marincola FM Expanding the perspective of translational medicine: the value of observational data J Transl Med 2012 10 61 10.1186/1479-5876-10-61 22452969 PMC3320561

[B5] Yan YX Liu YQ Li M Hu PF Guo AM Yang XH Development and evaluation of a questionnaire for measuring suboptimal health status in urban Chinese J Epidemiol 2009 19 6 333 341 10.2188/jea.JE20080086 19749497 PMC3924103

[B6] Yan YX Dong J Liu YQ Yang XH Li M Shia G Association of suboptimal health status and cardiovascular risk factors in urban chinese workers J Urban Health 2012 89 2 329 338 10.1007/s11524-011-9636-8 22203493 PMC3324604

[B7] Lu JP Knezevic A Wang YX Rudan I Campbell H Zou ZK Screening novel biomarkers for metabolic syndrome by profiling human plasma N-glycans in Chinese Han and Croatian populations J Proteome Res 2011 10 11 4959 4969 10.1021/pr2004067 21939225

[B8] Qin S Zhang C MicroRNAs in vascular disease J Cardiovasc Pharmacol 2011 57 1 8 12 10.1097/FJC.0b013e318203759b 21052012 PMC4517184

[B9] Ji R Cheng Y Yue J Yang J Liu X Chen H MicroRNA expression signature and antisense-mediated depletion reveal an essential role of MicroRNA in vascular neointimal lesion formation Circ Res 2007 100 11 1579 1588 10.1161/CIRCRESAHA.106.141986 17478730

[B10] Zhang C Novel functions for small RNA molecules Curr Opin Mol Ther 2009 11 6 641 651 20072941 PMC3593927

[B11] Cheng Y Tan N Yang J Liu X Cao X He P A translational study of circulating cell-free microRNA-1 in acute myocardial infarction Clin Sci (Lond) 2010 119 2 87 95 10.1042/CS20090645 20218970 PMC3593815

[B12] Cho WC Chow AS Au JS MiR-145 inhibits cell proliferation of human lung adenocarcinoma by targeting EGFR and NUDT1 RNA Biol 2011 8 1 125 131 10.4161/rna.8.1.14259 21289483

[B13] Cho WC Circulating MicroRNAs as Minimally Invasive Biomarkers for Cancer Theragnosis and Prognosis Front Genet 2011 2 7 22303306 10.3389/fgene.2011.00007 PMC3268566

[B14] Cho WC Proteomics and translational medicine: molecular biomarkers for cancer diagnosis, prognosis and prediction of therapy outcome Expert Rev Proteomics 2011 8 1 1 4 10.1586/epr.10.108 21329422

[B15] Fehniger TE Marko-Varga GA Clinical proteomics today J Proteome Res 2011 10 1 3 10.1021/pr1012569 21210716

[B16] Roepstorff P Fohlman J Proposal for a common nomenclature for sequence ions in mass spectra of peptides Biomed Mass Spectrom 1984 11 11 601 10.1002/bms.1200111109 6525415

[B17] Lopez E Wesselink JJ Lopez I Mendieta J Gomez-Puertas P Munoz SR Technical phosphoproteomic and bioinformatic tools useful in cancer research J Clin Bioinforma 2011 1 26 10.1186/2043-9113-1-26 21967744 PMC3195713

[B18] Lopez E Lopez I Sequi J Ferreira A Discovering and validating unknown phospho-sites from p38 and HuR protein kinases in vitro by Phosphoproteomic and Bioinformatic tools J Clin Bioinforma 2011 1 1 16 10.1186/2043-9113-1-16 21884634 PMC3164609

[B19] Moffatt MF Gut IG Demenais F Strachan DP Bouzigon E Heath S A large-scale, consortium-based genomewide association study of asthma N Engl J Med 2010 363 13 1211 1221 10.1056/NEJMoa0906312 20860503 PMC4260321

[B20] Vercelli D Discovering susceptibility genes for asthma and allergy Nat Rev Immunol 2008 8 3 169 182 10.1038/nri2257 18301422

[B21] Strachan DP Cook DG Health effects of passive smoking. 6. Parental smoking and childhood asthma: longitudinal and case–control studies Thorax 1998 53 3 204 212 10.1136/thx.53.3.204 9659358 PMC1745164

[B22] Ege MJ Strachan DP Cookson WO Moffatt MF Gut I Lathrop M Gene-environment interaction for childhood asthma and exposure to farming in Central Europe J Allergy Clin Immunol 2011 127 1 138 144 144 10.1016/j.jaci.2010.09.041 21211648

[B23] Hung RJ McKay JD Gaborieau V Boffetta P Hashibe M Zaridze D A susceptibility locus for lung cancer maps to nicotinic acetylcholine receptor subunit genes on 15q25 Nature 2008 452 7187 633 637 10.1038/nature06885 18385738

[B24] Kidwell CS Liebeskind DS Starkman S Saver JL Trends in acute ischemic stroke trials through the 20th century Stroke 2001 32 6 1349 1359 10.1161/01.STR.32.6.1349 11387498

[B25] Su Z Huang Y Zhou Q Wu Z Wu X Zheng Q High-level expression and purification of human epidermal growth factor with SUMO fusion in Escherichia coli Protein Pept Lett 2006 13 8 785 792 10.2174/092986606777841280 17073723

[B26] Wu X Su Z Li X Zheng Q Huang Y Yuan H High-level expression and purification of a nonmitogenic form of human acidic fibroblast growth factor in Escherichia coli Protein Expr Purif 2005 42 1 7 11 10.1016/j.pep.2004.07.021 15882952

[B27] Huang Z Zheng Q Wu X Su Z Xu H Tan Y Enhanced protection of modified human acidic fibroblast growth factor with polyethylene glycol against ischemia/reperfusion-induced retinal damage in rats Toxicol Lett 2007 170 2 146 156 10.1016/j.toxlet.2007.03.001 17416472

[B28] Huang Z Wang H Lu M Sun C Wu X Tan Y A better anti-diabetic recombinant human fibroblast growth factor 21 (rhFGF21) modified with polyethylene glycol PLoS One 2011 6 6 e20669 10.1371/journal.pone.0020669 21673953 PMC3108960

[B29] Vegvari A Welinder C Lindberg H Fehniger TE Marko-Varga G Biobank resources for future patient care: developments, principles and concepts J Clin Bioinforma 2011 1 1 24 10.1186/2043-9113-1-24 21923917 PMC3197484

[B30] Ansari D Rosendahl A Elebro J Andersson R Systematic review of immunohistochemical biomarkers to identify prognostic subgroups of patients with pancreatic cancer Br J Surg 2011 98 8 1041 1055 10.1002/bjs.7574 21644238

[B31] Cho WC Targeting the signaling pathways in cancer therapy Expert Opin Ther Targets 2012 16 1 1 3 22239441 10.1517/14728222.2011.648618

[B32] Almhanna K Philip PA Defining new paradigms for the treatment of pancreatic cancer Curr Treat Options Oncol 2011 12 2 111 125 10.1007/s11864-011-0150-8 21461670

[B33] Bossuyt PM Reitsma JB Bruns DE Gatsonis CA Glasziou PP Irwig LM Towards complete and accurate reporting of studies of diagnostic accuracy: the STARD initiative Clin Biochem 2003 36 1 2 7 12554053 10.1016/s0009-9120(02)00443-5

[B34] McShane LM Altman DG Sauerbrei W Taube SE Gion M Clark GM REporting recommendations for tumour MARKer prognostic studies (REMARK) Br J Cancer 2005 93 4 387 391 10.1038/sj.bjc.6602678 16106245 PMC2361579

[B35] Fehniger TE Vegvari A Rezeli M Prikk K Ross P Dahlback M Direct demonstration of tissue uptake of an inhaled drug: proof-of-principle study using matrix-assisted laser desorption ionization mass spectrometry imaging Anal Chem 2011 83 21 8329 8336 10.1021/ac2014349 21942412

